# Environmental Persistence of Influenza Viruses Is Dependent upon Virus Type and Host Origin

**DOI:** 10.1128/mSphere.00552-19

**Published:** 2019-08-21

**Authors:** Karen A. Kormuth, Kaisen Lin, Zhihong Qian, Michael M. Myerburg, Linsey C. Marr, Seema S. Lakdawala

**Affiliations:** a Department of Microbiology & Molecular Genetics, University of Pittsburgh School of Medicine, Pittsburgh, Pennsylvania, USA; b Department of Biology, Bethany College, Bethany, West Virginia, USA; c Department of Civil and Environmental Engineering, Virginia Tech, Blacksburg, Virginia, USA; d Tsinghua University School of Medicine, Beijing, China; e Department of Medicine, Division of Pulmonary, Allergy, and Critical Care Medicine, University of Pittsburgh School of Medicine, Pittsburgh, Pennsylvania, USA; f Center for Vaccine Research, University of Pittsburgh School of Medicine, Pittsburgh, Pennsylvania, USA; Emory University School of Medicine

**Keywords:** aerosols, droplets, influenza virus, persistence, relative humidity

## Abstract

Highly transmissible influenza viruses (IV) must remain stable and infectious under a wide range of environmental conditions following release from the respiratory tract into the air. Understanding how expelled IV persist in the environment is critical to limiting the spread of these viruses. Little is known about how the stability of different IV in expelled aerosols is impacted by exposure to environmental stressors, such as relative humidity (RH). Given that not all IV are equally capable of efficient airborne transmission in people, we anticipated that not all IV would respond uniformly to ambient RH. Therefore, we have examined the stability of human-pathogenic seasonal and avian IV in suspended aerosols and stationary droplets under a range of RH conditions. H3N2 and influenza B virus (IBV) isolates are resistant to RH-dependent decay in aerosols in the presence of human airway surface liquid, but we observed strain-dependent variations in the longevities of H1N1, H3N2, and IBV in droplets. Surprisingly, low-pathogenicity avian influenza H6N1 and H9N2 viruses, which cause sporadic infections in humans but are unable to transmit person to person, demonstrated a trend toward increased sensitivity at midrange to high-range RH. Taken together, our observations suggest that the levels of vulnerability to decay at midrange RH differ with virus type and host origin.

**IMPORTANCE** The rapid spread of influenza viruses (IV) from person to person during seasonal epidemics causes acute respiratory infections that can lead to hospitalizations and life-threatening illness. Atmospheric conditions such as relative humidity (RH) can impact the viability of IV released into the air. To understand how different IV are affected by their environment, we compared the levels of stability of human-pathogenic seasonal and avian IV under a range of RH conditions and found that highly transmissible seasonal IV were less sensitive to decay under midrange RH conditions in droplets. We observed that certain RH conditions can support the persistence of infectious viruses on surfaces and in the air for extended periods of time. Together, our findings will facilitate understanding of factors affecting the persistence and spread of IV in our environment.

## Abstract (precis)

The rapid spread of influenza viruses (IV) from person to person during seasonal epidemics causes acute respiratory infections that can lead to hospitalizations and life-threatening illness. Atmospheric conditions such as relative humidity (RH) can impact the viability of IV released into the air. To understand how different IV are affected by their environment, we compared the levels of stability of human-pathogenic seasonal and avian IV under a range of RH conditions and found that highly transmissible seasonal IV were less sensitive to decay under midrange RH conditions in droplets. We observed that certain RH conditions can support the persistence of infectious viruses on surfaces and in the air for extended periods of time. Together, our findings will facilitate understanding of factors affecting the persistence and spread of IV in our environment.

## INTRODUCTION

Seasonal influenza viruses (IV) cause yearly infection cycles that tend to peak in the winter in temperate regions and during rainy periods in tropical climates (1). Seasonal IV epidemics create a significant public health burden, with the 2017-to-2018 season resulting in the highest number of deaths since 2010 and >30,000 hospitalizations in the United States alone (2). Seasonal variation in environmental parameters, including relative humidity (RH), has been implicated in the seasonal spread of these viruses (3–5). Efficient epidemiological spread of IV in people is dictated by the capacity of the viruses to transmit effectively through the air within respiratory aerosols (noncontact transmission) and droplets (indirect contact, or fomite transmission) expelled from an infected host (6). These modes of transmission require that the viruses maintain viability in the environment for the period of time leading up to contact with an immunologically naive recipient. However, little is known about the maintenance and duration of viral stability in the environment following release from the human airway. Clarifying the relationship between RH and viral persistence in the environment will be critical to understanding the basis for seasonal epidemiology of IV, as well as to designing nonpharmaceutical intervention strategies to limit the spread of these viruses in the human population.

Variations in ambient RH have previously been shown to directly affect transmission of IV in animal models (7, 8). Historically, midrange RH conditions have been shown to be detrimental to the viability of expelled IV (4, 9–14). However, our recent work has demonstrated that the presence of airway surface liquid (ASL) collected from human bronchial epithelial (HBE) cells can protect the 2009 H1N1 pandemic (H1N1pdm) virus from RH-dependent decay in suspended aerosols and stationary droplets (15). Primary HBE cells differentiated at an air-liquid interface produce mucus, mimic the surface of the lumen of the human airway, and are highly permissive to IV infection (16, 17). Newly replicated IV collected from the apical surface of HBE cells are, therefore, expected to be very similar to those expelled in physiological respiratory droplets. Comparing the levels of stability of IV under a range of RH conditions in physiologically relevant aerosols and droplets will provide a better representation of how IV respond to environmental stressors following release from the respiratory tract and will improve assessment of the risk of transmission under different environmental conditions during an influenza epidemic.

Recurring epidemics are driven by cocirculation of human-pathogenic seasonal H1N1 and H3N2 subtype influenza A viruses (IAV) and influenza B viruses (IBV), which persist in the population despite significant surveillance and intervention efforts (18). The epidemiological spread of IAV and that of IBV are not uniform, with IAV and IBV infections peaking at different times during a season (https://www.cdc.gov/flu/weekly/index.htm) (19–21), which may reflect differences in their primary modes of transmission (22). The impact of the environment on the seasonality of influenza viruses in temperate climates is still poorly understood. One approach to addressing this critical issue is that of comparing the environmental persistence of IV that transmit efficiently through the air (i.e., human-pathogenic seasonal viruses) to that of IV that are poorly transmissible in the human population. Low-pathogenicity avian influenza (LPAI) viruses, including H6N1 and H9N2, have caused sporadic infections in humans (23–26) but fail to transmit efficiently in animal models (27–30). Previous pandemic IV have emerged through genetic reassortment of seasonal and zoonotic IAV (31, 32). Therefore, comparisons of the levels of environmental persistence of human IV and LPAI viruses will improve our understanding of the contribution of environmental factors to the spread of IV in the human population and enhance pandemic preparedness programs that assess the phenotypes of emerging zoonotic IAV with pandemic potential.

In this study, we examined the environmental persistence of six IV, including human-pathogenic seasonal and LPAI viruses (Table 1; see also Table S1 in the supplemental material), in response to a range of RH conditions. We explored the contributions of virus strain background, droplet composition via various propagation methods, RH, and duration of exposure on the stability of IV in aerosols and large droplets. We found that human-pathogenic seasonal H3N2 and IBV were resistant to decay under most RH conditions for an extended period of time in aerosols containing ASL from HBE cells (HBE ASL). However, the longevities of human-pathogenic seasonal IV stability in droplets differed between subtypes H1N1, H3N2, and IBV in an RH-dependent manner, suggesting a role for virus-specific factors in the environmental persistence of IV. Surprisingly, we observed that LPAI IV were more vulnerable to decay at midrange RH than human-pathogenic seasonal IV. Together, these results confirm that human-pathogenic seasonal IV can remain infectious under a range of RH conditions, in agreement with our previous study (15), but this work clearly demonstrates that RH may be an important factor affecting the stability of expelled IV over time. Overall, our results indicate that the levels of persistence of IV are not uniform and that virus-specific factors can impact the stability and longevity of IV in the environment.

**TABLE 1:** Influenza virus strains used in this study

Type	Strain	Identifier
IAV	A/CA/07/2009 (H1N1)	H1N1pdm
IAV	A/Brisbane/59/2007 (H1N1)	Bris H1N1
IAV	A/Perth/16/09 (H3N2)	Perth H3N2
IBV	B/Texas/02/2013	IBV
IAV	A/Shorebird/DE Bay/230/2009 (H6N1)	avH6N1
IAV	A/Shorebird/DE/127/2003 (H9N2)	avH9N2

10.1128/mSphere.00552-19.9 TABLE S1 Influenza virus passage history and references. Download Table S1, DOCX file, 0.01 MB. Copyright © 2019 Kormuth et al. 2019 Kormuth et al. This content is distributed under the terms of the Creative Commons Attribution 4.0 International license.

## RESULTS

### Aerosolized seasonal H3N2 and IBV are stable independently of RH.

Recently, we demonstrated that the presence of airway surface liquid (ASL), collected from HBE cells grown at an air-liquid interface, confers a protective effect against RH-dependent decay to H1N1pdm virus grown in MDCK (Madin-Darby canine kidney) cells in aerosols and droplets (15). H1N1pdm viruses grown in HBE cells are also protected from decay under midrange RH conditions (15). These data suggest that HBE ASL provides a protective microenvironment for H1N1pdm. To determine whether this effect is consistent among other seasonal IV, we compared the levels of stability of aerosolized Perth H3N2 and IBV in the presence of HBE ASL.

**FIG 1:**
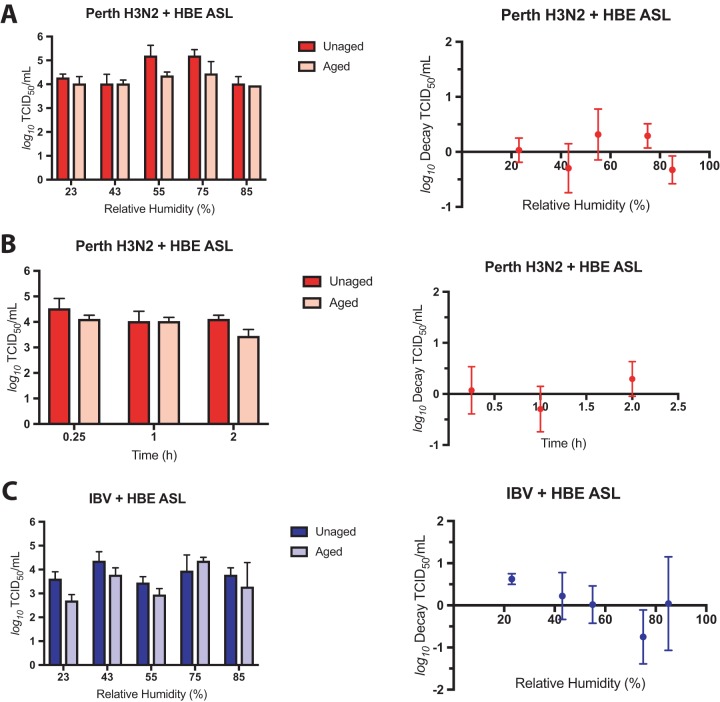
Aerosolized seasonal IV are stable under a range of RH conditions. MDCK cell-grown Perth H3N2 or IBV supplemented with ASL collected from uninfected HBE cells were aerosolized into a rotating drum under five different sets of RH conditions. Infectious titers were determined in unaged and aged aerosol samples (left), and decay was quantified as the difference (right), corrected for losses in the drum. (A) Perth H3N2 plus HBE ASL did not decay after 1 h in aerosols under all five RH conditions tested. (B) Aerosolized Perth H3N2 plus HBE ASL remained equally stable after 15 min, 1 h, or 2 h at 43% RH. (C) Aerosolized IBV plus HBE ASL was highly stable under all RH conditions tested, with some (<1 log_10_) decay occurring at 23% RH, although the level of decay was not significantly different from that seen with Perth H3N2 under any RH condition. Data represent mean values ± standard deviations of the results from all of the experiments, each of which was conducted in triplicate.

To test the stability of other seasonal IV in aerosols, we aerosolized Perth H3N2 or IBV supplemented with a 1:10 dilution of HBE ASL collected from uninfected cells into a rotating, RH-controlled drum. Changes in infectious virus titer were determined by comparison of TCID_50_ (50% tissue culture infectious dose) assay results between unaged aerosols and aerosols aged for 1 h in the drum determined on MDCK cells. Log decay was corrected for both physical loss and dilution in the drum at each RH (see Fig. S1 in the supplemental material). The titer of Perth H3N2 did not change between aged and unaged samples in the presence of HBE ASL, and we did not detect any RH-dependent decay of Perth H3N2 in aerosols after 1 h (Fig. 1A). To assess the persistence of aerosolized Perth H3N2, we aged viral aerosols in the presence of HBE ASL for 15 min, 1 h, and 2 h at 43% RH. We found that the virus remained fully infectious in aerosols under those conditions for up to 2 h (Fig. 1B). Similarly, aerosolized IBV remained equally infectious using unaged and aged aerosols at 43%, 55%, 75%, and 95% RH for up to 1 h in the drum (Fig. 1C). These results indicate that aerosolized human-pathogenic seasonal IV can resist RH-dependent decay and are more persistent in the environment than previously suggested with laboratory-adapted strains of IAV (4, 9–14).

10.1128/mSphere.00552-19.1 FIG S1 Physical loss (*k_p_*) and dilution (*k_d_*) coefficients under each set of RH conditions in the rotating drum and confirmation of virus conservation in the drum. Physical loss and dilution coefficients were determined following measurement of aerosol volume concentration using scanning mobility and aerodynamic particle sizers. Coefficients were determined by fitting the data to a first-order decay model. (A) *k_p_* and *k_d_* determined under each set of RH conditions using betapropiolactone (BPL)-inactivated Perth H3N2 plus ASL from HBE cells. (B) *k_p_* and *k_d_* determined under each set of RH conditions for BPL-inactivated IBV plus ASL from HBE cells. (C) To ensure that similar amounts of viral material were collected before and after aging during the temporal experiment performed with Perth H3N2, we performed qPCR on samples before and after aging for 15 min, 1 h, and 2 h. Data are representative of results from three replicate experiments (denoted by symbols), where viral aerosols were sampled independently three times from the RH drum. Bars represent means ± standard deviations. Download FIG S1, EPS file, 2.2 MB. Copyright © 2019 Kormuth et al. 2019 Kormuth et al. This content is distributed under the terms of the Creative Commons Attribution 4.0 International license.

### Cell propagation method impacts stability of H1N1 but not H3N2 viruses in stationary droplets.

**FIG 2:**
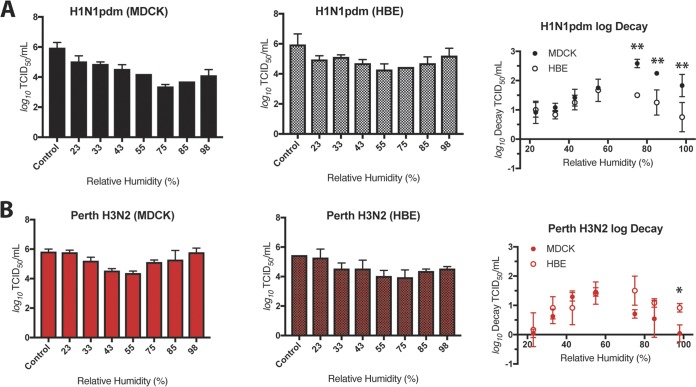
Decay of seasonal IAV in droplets depends on virus type and propagation method. H1N1pdm and Perth H3N2 were propagated in MDCK cells or HBE cells. Virus-containing droplets were incubated under seven different RH conditions for 2 h, and infectious titers were determined by TCID_50_ assay. Viral titers of MDCK-grown virus are shown in the left graph, HBE-grown virus in the center graph, and log decay of both MDCK- and HBE-grown virus in the right graph of each panel. (A) The titers of both MDCK cell- and HBE cell-grown H1N1pdm viruses varied with RH, with the most severe loss in titer occurring at 75%, 85%, and 98% RH for the MDCK cell-grown stock. In contrast, HBE cell-grown H1N1pdm was protected from decay at mid-to-high-RH conditions. (B) Titers of both MDCK cell- and HBE cell-grown Perth H3N2 viruses varied with RH, with the greatest effect observed at midrange RH. Unlike the result seen with H1N1pdm, the cell propagation method did not affect the level of sensitivity to RH, with one exception seen at 98% RH. Data are presented as mean values ± standard deviations of the results from all of the experiments, each of which was conducted in triplicate, and are representative of at least two biological replicate experiments. Asterisks indicate significantly different mean levels of decay between MDCK cell- and HBE cell-grown viruses under each set of conditions, as determined by two-way ANOVA, and adjusted *P* values are reported using Bonferroni’s correction for multiple comparisons (*, *P* < 0.05; **, *P* < 0.01).

To assess the stability of human-pathogenic seasonal IV in large droplets, we used 1-μl droplets of viruses grown in MDCK or HBE cells under seven sets of RH conditions: 23%, 33%, 43%, 55%, 75%, 85%, and 98% RH for 2 h. After this incubation period, infectious viral titer was determined by TCID_50_ assay on MDCK cells and compared to the titers determined using an equivalent volume (10 μl total) of virus incubated in a sealed vial outside the chamber. The levels of viral stability determined under each set of RH conditions are presented as the raw viral titer and log decay, which is the change in infectivity between experimental and control samples. Consistent with our previous findings (15), we found that H1N1pdm is highly sensitive to midrange RH under conditions of propagation in MDCK cells but is less sensitive to RH following propagation in HBE cells (Fig. 2A). Specifically, we observed a decrease of >2 log_10_ of virus titer for MDCK cell-grown H1N1pdm at 75% and 85% RH compared to control samples. In contrast, H1N1pdm propagated in HBE cells was resistant to RH-mediated decay. Comparisons between the level of decay determined for H1N1pdm grown in MDCK cells and that grown in HBE cells revealed a statistically significant difference in viral decay levels for HBE cell-propagated H1N1pdm compared to the MDCK cell-grown virus at 75%, 85%, and 98% RH, suggesting that growth in HBE cells enhances the stability of H1N1pdm under these RH conditions. To account for effects of patient-specific variation on IV stability, we propagated the viruses in multiple HBE cell cultures derived from different patient tissue samples for our experiments (see Table S2 in the supplemental material). We found no significant differences in the levels of decay of H1N1pdm in any of the three primary HBE cell cultures tested (Fig. S2).

10.1128/mSphere.00552-19.2 FIG S2 Sensitivity of H1N1pdm to RH in stationary droplets following growth in HBE cells. H1N1pdm was grown in primary HBE cell lines derived from 3 different patient samples. Droplets containing virus were incubated under a range of RH conditions for 2 h. Levels of decay of the virus were compared among the three HBE cell lines, and the results were not significantly different, as determined by one-way ANOVA with Tukey’s multiple-comparison test. Data are presented as mean values ± standard deviations of the results from all of the experiments, each of which was conducted in triplicate. Download FIG S2, EPS file, 1.9 MB. Copyright © 2019 Kormuth et al. 2019 Kormuth et al. This content is distributed under the terms of the Creative Commons Attribution 4.0 International license.

10.1128/mSphere.00552-19.10 TABLE S2 Influenza viruses and corresponding HBE cell cultures used for RH chamber experiments. Download Table S2, DOCX file, 0.01 MB. Copyright © 2019 Kormuth et al. 2019 Kormuth et al. This content is distributed under the terms of the Creative Commons Attribution 4.0 International license.

To examine whether H1N1pdm decay was representative of that of other human H1N1 viruses, we assessed the decay of a pre-2009 pandemic seasonal H1N1 (Bris H1N1) strain (Fig. S3). Similarly to the results seen with H1N1pdm, we found that the levels of stability of BrisH1N1 were statistically significantly different between MDCK and HBE cells at 43%, 55%, and 75% RH. These data indicate that sensitivity of H1N1 viruses to RH in the absence of HBE ASL is not limited to viruses emerging after the 2009 pandemic. In contrast, analysis of the stability of Perth H3N2 IV in stationary droplets showed that the levels were not significantly different in comparisons between cell propagation methods at all RH settings, excluding 98%, where HBE-grown H3N2 decayed more than MDCK-grown H3N2 (Fig. 2B; see also Fig. S4). In both cases, Perth H3N2 showed a trend toward more decay at midrange RH (43% to 75%) than at low or high RH. Our data suggest that viral propagation method impacts the stability of H1N1 viruses more than H3N2 viruses, at least for those isolates analyzed in this study, indicating that IV do not all respond to environmental stimuli in the same manner.

10.1128/mSphere.00552-19.3 FIG S3 Pre-2009 A/Brisbane/59/2007, Bris H1N1, is protected from RH following growth in HBE cells. Bris H1N1 viruses grown in MDCK and HBE cells were exposed to seven different sets of RH conditions for 2 h in droplets. The infectious titer of the MDCK cell-grown virus showed that it was more sensitive to midrange RH than the HBE cell-grown stock, with significantly less decay of the HBE virus at 43%, 55%, and 75% RH. Data are presented as mean values ± standard deviations of the results from all of the experiments, each of which was conducted in triplicate, using virus from a single HBE cell line. Asterisks indicate significantly different mean levels of log decay over time, as determined by two-way ANOVA, and adjusted *P* values are reported using Bonferroni’s correction for multiple comparisons (*, *P* < 0.05; **, *P* < 0.01). Download FIG S3, EPS file, 2 MB. Copyright © 2019 Kormuth et al. 2019 Kormuth et al. This content is distributed under the terms of the Creative Commons Attribution 4.0 International license.

10.1128/mSphere.00552-19.4 FIG S4 Sensitivity of HBE cell-grown Perth H3N2 in stationary droplets. Perth H3N2 was grown in primary HBE cell lines derived from 3 different patient samples. Droplets containing virus were incubated under a range of RH conditions for 2 h. Levels of decay of the virus were compared among three HBE cell lines and were not significantly different among the three HBE cell lines, as determined by one-way ANOVA with Tukey’s multiple-comparison test. Data are presented as mean values ± standard deviations of the results from all of the experiments, each of which was conducted in triplicate. Download FIG S4, EPS file, 1.9 MB. Copyright © 2019 Kormuth et al. 2019 Kormuth et al. This content is distributed under the terms of the Creative Commons Attribution 4.0 International license.

### Seasonal IBV is sensitive to RH-mediated decay in stationary droplets.

**FIG 3:**
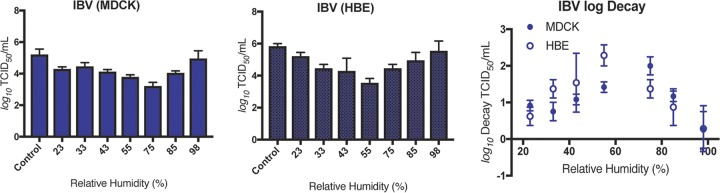
Seasonal IBV is sensitive to RH in droplets. MDCK cell- and HBE cell-propagated IBV was exposed to seven different sets of RH conditions in droplets for 2 h. Viral titers of MDCK-grown virus are shown in the left graph, HBE-grown virus in the center graph, and log decay of both MDCK- and HBE-grown virus in the right graph. Decay levels of MDCK cell- and HBE cell-grown IBV were not significantly different under any RH condition tested, as determined by two-way ANOVA. Data are presented as mean values ± standard deviations of the results from all of the experiments, each of which was conducted in triplicate, and are representative of at least two biological replicate experiments.

Seasonal influenza infections are caused by IAV subtypes H1N1 and H3N2 and by IBV (18). However, in contrast to IAV, the onset and spread of IBV within the community tends to occur primarily in children (33), with an estimated efficiency of transmission in households of <40% by the aerosol route (22). To test the effect of RH on IBV in droplets, a strain from the Victoria lineage was grown in MDCK and HBE cells. This virus replicated to high titers in HBE cells (Fig. S5A). MDCK cell-grown IBV stability varied with RH, with the greatest average decay (>2 log_10_) occurring at 75% RH (Fig. 3). Surprisingly, HBE cell-grown IBV also decayed >2 log_10_ at a midrange RH (55%). No significant difference in log decay was observed between the two propagation methods, suggesting that HBE ASL does not protect IBV from sensitivity to RH. This phenotype was reproduced in two additional HBE cultures derived from distinct patient samples (Fig. S5B). The decay of IBV did not match its resistance to RH in aerosols, which may suggest a role for aerosolization mechanism or droplet size in resistance to RH-dependent decay, although the duration of the incubation for aerosols was half as long as for droplets. The decay of HBE cell-grown IBV was significantly greater (two-way analysis of variance [ANOVA] with Bonferroni’s multiple-comparison test) than that of HBE cell-grown Perth H3N2 at 55% RH. In addition, we did not observe a >2 log_10_ decay for H1N1pdm or Perth H3N2 viruses grown in HBE cells under any RH condition. These results, as well as our observation that there was not a significant difference in decay between MDCK cell-grown IBV and HBE cell-grown IBV, in contrast to our observations with H1N1pdm, suggest that IAV and IBV isolates may respond differently to RH in droplets. Such differences in vulnerability to RH-medicated decay may be linked to the differential seasonal infection cycles of these viruses in nature.

10.1128/mSphere.00552-19.5 FIG S5 Sensitivity of seasonal IBV in stationary droplets under conditions of virus growth in HBE cells. (A) Replication kinetics of IBV in HBE cells demonstrates efficient replication of the virus in a primary cell line. Data are shown as means ± standard deviations of values from three transwells representing TCID_50_ per milliliter. (B) IBV was grown in primary HBE cell lines derived from 3 different patient samples. Droplets containing virus were incubated under a range of RH conditions for 2 h. Levels of decay of the virus were not significantly different among the three HBE cell lines, as determined by one-way ANOVA with Tukey’s multiple-comparison test. Data are presented as mean values ± standard deviations of the results from all of the experiments, each of which was conducted in triplicate. Download FIG S5, EPS file, 1.9 MB. Copyright © 2019 Kormuth et al. 2019 Kormuth et al. This content is distributed under the terms of the Creative Commons Attribution 4.0 International license.

### Longevity of seasonal IV in droplets is dependent upon RH and virus strain.

**FIG 4:**
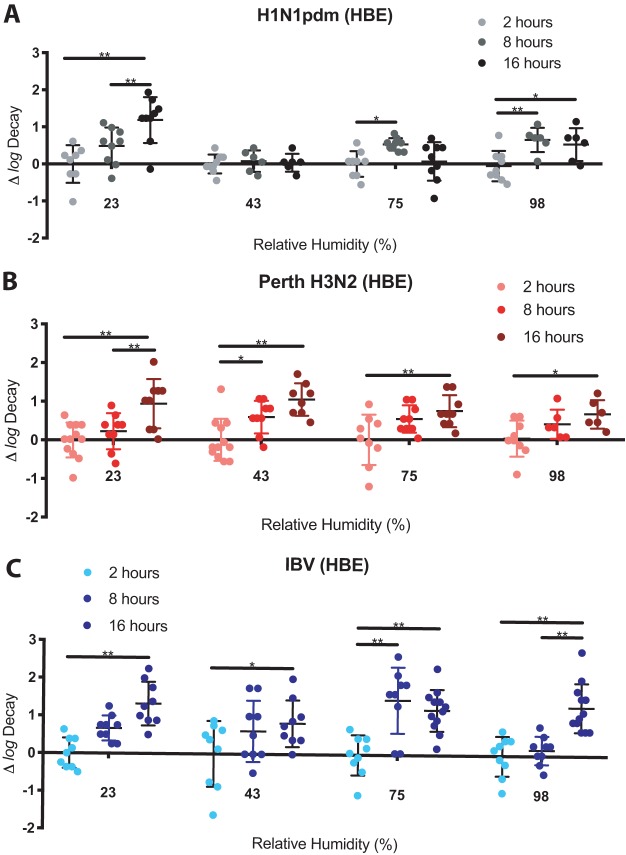
The rate of decay of seasonal IV depends upon both RH and virus strain background. Droplets containing HBE cell-grown H1N1pdm, Perth H3N2, or IBV were exposed to four different sets of RH conditions for 2, 8, and 16 h. The rate of decay (Δ log decay) was quantified as the difference of the log decay level at each RH level from the mean log decay level at 2 h for each virus. (A) The rate of decay of H1N1pdm increased significantly from 2 to 16 h at 23% and 98% RH. However, at 43% and 75% RH, the Δ log decay remained fixed for up to 16 h, indicating that the virus was highly stable under these conditions. (B and C) Δ log decay of HBE cell-grown Perth H3N2 (B) and IBV (C) increased significantly from 2 to 16 h under all four sets of RH conditions tested, indicating increased sensitivity to RH over time compared to H1N1pdm. Data were compiled from results determined for viruses grown in at least 3 HBE cell lines and are presented as individual values (symbols). Error bars represent means ± standard deviations. Asterisks indicate significantly different mean levels of log decay over time, as determined by two-way ANOVA, and adjusted *P* values are reported using Bonferroni’s correction for multiple comparisons (*, *P* < 0.05; **, *P* < 0.01).

The risk of infection by indirect contact, or by fomite transmission, of a seasonal IV depends on the ability of the viruses to remain infectious for a long period of time following deposition onto a surface. To understand the persistence of seasonal IV in droplets, we tested the stability of HBE cell-propagated H1N1pdm, Perth H3N2, and IBV after 2, 8, and 16 h under four different sets of RH conditions: 23%, 43%, 75%, and 98%. We confirmed that the RH chamber was capable of maintaining constant temperature and humidity for the duration of our studies (Fig. S6). The rate of change in virus titer, presented as (Δ log decay), was determined as the difference between the decay at each RH and the mean log decay at 2 h for each virus. We performed each study in three independent biological replicates from three different patient cell lines (Table S2). Each replicate is presented to illustrate the variation observed under each set of RH conditions over time (Fig. 4). In general, we anticipated that all viruses would decay over time but that the relative amounts of decay would depend upon both virus strain and RH.

10.1128/mSphere.00552-19.6 FIG S6 RH chambers maintain stable RH and temperature over long incubation times. Longitudinal virus stability assays require extended incubations in the RH chamber. (A) Stable RH was maintained beyond 16 h for each set of RH conditions tested. Brief deviations in RH resulted from the chamber being opened to allow placement of the samples. (B) Stable temperature within the chambers was maintained beyond 16 h for each set of RH conditions tested. Download FIG S6, EPS file, 2.3 MB. Copyright © 2019 Kormuth et al. 2019 Kormuth et al. This content is distributed under the terms of the Creative Commons Attribution 4.0 International license.

Surprisingly, the Δ log decay of H1N1pdm in droplets was highly variable based on RH condition (Fig. 4A). Initial average decay at 2 h was determined on the basis of the data presented in Fig. 2 (H1N1pdm and Perth H3N2) and Fig. 3 (IBV). Most strikingly, the infectious titer of HBE cell-propagated H1N1pdm did not diminish at 43% RH, suggesting that the virus was capable of remaining fully infectious in droplets under those conditions for up to 16 h. At 23% and 98% RH, the Δ log decay of H1N1pdm increased from 2 to 16 h. However, at 75% RH, H1N1pdm Δ log decay was different from 2 to 8 h but not 2 to 16 h. We observed a more consistent loss of infectious titer over time for Perth H3N2 and IBV than for H1N1pdm. Specifically, the Δ log decay of Perth H3N2 increased significantly between 2 and 16 h under all four RH conditions tested (Fig. 4B). Similarly, Δ log decay of HBE cell-grown IBV also increased significantly from 2 to 16 h under all four RH conditions tested (Fig. 4C). Mixed-effects analyses using Tukey’s multiple-comparison test confirmed that Δ log decay of H1N1pdm was significantly different from that of both Perth H3N2 (*P* < 0.01) and IBV (*P* < 0.05) at 43% RH as well as from that of IBV (*P* < 0.01) at 75% RH. Δ log decay of Perth H3N2 was significantly different from that of IBV (*P* < 0.05) at 75% by the same statistical analysis.

Together, these data indicate that the levels of persistence of seasonal IV in droplets differ among virus types and subtypes but also that IV can remain stable and highly infectious for long periods of time under certain RH conditions.

### RH-dependent decay of LPAI viruses in droplets.

**FIG 5:**
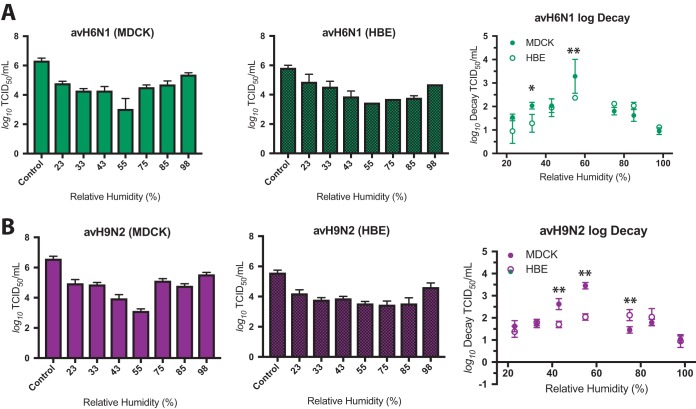
Sensitivity of LPAI to RH in stationary droplets. LPAI viruses were grown in MDCK or HBE cells, and virus-containing droplets were exposed to seven different sets of RH conditions for 2 h. Viral titers of MDCK-grown virus are shown in the left graph, HBE-grown virus in the center graph, and log decay of both MDCK- and HBE-grown virus in the right graph of each panel. (A) Both MDCK cell-grown avH6N1 and HBE cell-grown avH6N1 viruses were highly sensitive to RH, with mean decay levels exceeding 3 log_10_ at 55% RH. Decay was significantly attenuated at 33% and 55% RH in the HBE cell-grown stock compared to MDCK cell-grown virus. (B) MDCK cell-grown avH9N2 virus was similarly sensitive to midrange RH. Significantly less decay was observed for HBE cell-grown viruses at 43% and 55% RH, although we detected significantly more decay of avH9N2 (HBE) at 75% RH. Data are presented as mean values ± standard deviations of the results from all of the experiments, each of which was conducted in triplicate, and are representative of at least two biological replicate experiments. Asterisks indicate significantly different mean levels of decay over time as determined by two-way ANOVA, and adjusted *P* values are reported using Bonferroni’s correction for multiple comparisons (*, *P* < 0.05; **, *P* < 0.01).

To this point, our study had focused on the persistence of epidemiologically successful human-pathogenic seasonal IV. However, the host range of IAV is quite broad, resulting in the emergence of IAV pandemics from animal sources (27, 31). LPAI viruses, including H9N2 and H6N1 subtypes, have contributed to zoonotic infections (23–26) but have not yet spread efficiently through the human population (27). To understand how environmental factors may affect the spread of LPAI viruses, we tested the stability of MDCK cell-propagated and HBE cell-propagated avian influenza virus H6N1 (avH6N1) and avH9N2 strains in large droplets under a range of RH conditions. Both of the LPAI virus strains replicated well in HBE cells, with titers exceeding 10^7^ TCID_50_/ml (Fig. S7A). We hypothesized that, like the seasonal IAV reported here, the LPAI IAV would be resistant to RH-dependent decay following propagation in HBE cells. MDCK cell-grown avH6N1 and avH9N2 decayed >2 log_10_ at midrange RH (Fig. 5). The severity of decay of these viruses was greater for the MDCK cell-grown stock at 43% and 55% RH than for the HBE cell-grown viruses, but both reached at least 2 log_10_ decay at midrange RH, suggesting that these viruses are more sensitive to RH in droplets than the human-pathogenic seasonal H1N1pdm and Perth H3N2 viruses tested in this study. The RH sensitivity phenotypes of both avH6N1 and avH9N2 were reproduced in 2 additional HBE cell cultures, although avH6N1 grown in a third patient culture (HBE 0206) decayed significantly less than virus grown in HBE 0195 or HBE 0204 cells (Fig. S7B). These data underscore the need to assess viral stability in samples prepared in, at minimum, three primary cell lines to ensure reproducibility.

10.1128/mSphere.00552-19.7 FIG S7 LPAI viruses propagated in HBE cells are sensitive to RH-dependent decay in droplets. (A) Replication kinetics of avH6N1 (left) and avH9N2 (right) in HBE cells demonstrating that the avian viruses grow well in the human cell line. Data are shown as means ± standard deviations of values from three transwells representing the TCID_50_ per milliliter for each virus. (B) Decay of LPAI viruses grown in HBE cells. LPAI viruses were propagated in three distinct HBE cell cultures, and virus stability was determined following a 2-h exposure to 7 different sets of RH conditions. For both LPAI virus experiments, the level of decay of viruses grown in HBE 0206 was significantly different from that seen with viruses grown in the other two HBE cell lines (determined by two-way ANOVA using Bonferroni’s correction for multiple comparisons). Data are presented as mean values ± standard deviations of the results from all of the experiments, each of which was conducted in triplicate. Asterisks represent RH conditions where decay of virus grown in HBE 0206 was significantly different from that seen with either one (*) or both (**) of the other two HBE cell lines. Download FIG S7, EPS file, 2.2 MB. Copyright © 2019 Kormuth et al. 2019 Kormuth et al. This content is distributed under the terms of the Creative Commons Attribution 4.0 International license.

### avH6N1 is less stable at midrange RH than human-pathogenic seasonal IV.

**FIG 6:**
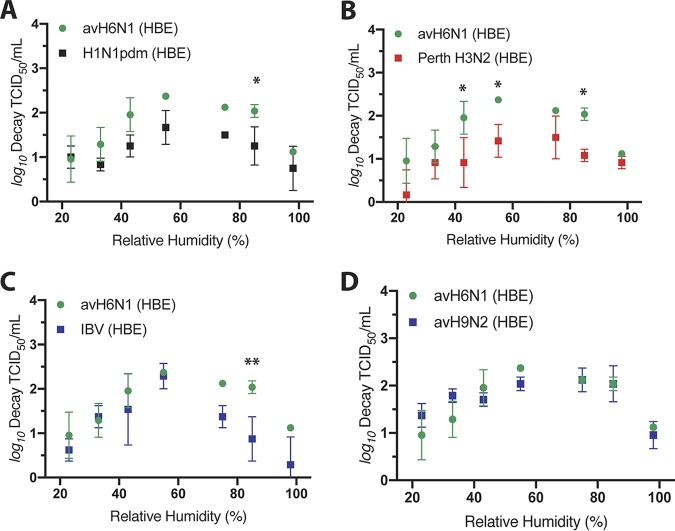
HBE cell-grown avH6N1 virus is more sensitive to RH than human-pathogenic seasonal IV in stationary droplets. To compare the stability levels of distinct IV isolates following growth in HBE cells, we reanalyzed the decay of avH6N1 compared to the other human-pathogenic seasonal and LPAI isolates used in this study. (A) The level of avH6N1 decay was significantly greater than that of H1N1pdm at 75% RH. (B) The level of avH6N1 decay was significantly greater than that of Perth H3N2 at 43%, 55%, and 85% RH. (C) The level of avH6N1 decay was significantly greater than that of IBV at 85% RH. (D) The decay levels of avH6N1 were not significantly different from those of avH9N2 at any RH tested. Significance was determined at each RH using a two-way ANOVA, and adjusted *P* values are reported using Bonferroni’s correction for multiple comparisons. Data represent means ± standard deviations of results of experiments conducted in triplicate (*, *P* < 0.05; **, *P* < 0.01).

In comparing the data from the different isolates tested in this study, we noticed that avH6N1 appeared to be highly sensitive to RH, reaching average decay levels above 3 log_10_ at 55% RH (Fig. 5A). To assess whether this virus decayed differently from the other viruses tested, we compared the levels of decay under each set of RH conditions for HBE cell-grown avH6N1 with H1N1pdm (Fig. 6A), Perth H3N2 (Fig. 6B), and IBV (Fig. 6C) as well as with the other LPAI virus, avH9N2 (Fig. 6D). We detected significantly more decay of avH6N1 than of each of the human-pathogenic seasonal IV for at least 1 midrange RH condition. In contrast, decay of avH6N1 was not significantly different from decay of avH9N2 under any RH condition, suggesting that this LPAI isolate may be less resistant to RH-dependent decay than the highly transmissible human-pathogenic seasonal viruses. Human and avian IV generally differ in hemagglutinin (HA) receptor specificity, with human IV preferentially binding to α2,6-linked sialic acids and avian IV preferring α2,3 sialic acids (34, 35). To test whether receptor specificity impacts virus stability at midrange RH, we compared the decay of wild-type H1N1pdm with that of a mutant H1N1pdm having preferential binding to α2,3-linked sialic acids (Fig. S8) that we had previously characterized (36). We found significantly more decay of the α2,3 HA H1N1pdm mutant under 43% RH conditions than of the wild-type viruses. This result was confirmed in three independent HBE cultures, indicating that viral persistence may be influenced, at least in part, by HA receptor binding preference.

10.1128/mSphere.00552-19.8 FIG S8 A mutant H1N1pdm virus with a preference for binding avian cell surface receptors is less stable than wild-type H1N1pdm. α2,3 H1N1pdm was grown in three primary HBE cell lines. Combined droplet decay levels of wild-type H1N1pdm grown in three HBE cell lines were compared to the level of decay seen with the mutant virus grown in three HBE cell lines. Decay of virus from each individual cell line was determined from experiments conducted in triplicate. Significantly more decay of α2,3 H1N1pdm was detected under 43% RH conditions by two-way ANOVA, and adjusted *P* values are reported using Bonferroni’s correction for multiple comparisons (**, *P* < 0.01). Symbols represent individual decay values, and error bars represent means ± standard deviations. Download FIG S8, EPS file, 2 MB. Copyright © 2019 Kormuth et al. 2019 Kormuth et al. This content is distributed under the terms of the Creative Commons Attribution 4.0 International license.

## DISCUSSION

With this work, we have produced a comprehensive investigation of the relationship between RH and the stability of seasonal IV and LPAI in the environment. We found that stability of airborne IV in aerosols and droplets is not unique to H1N1pdm as previously reported (15) but is a trait shared by other seasonal IV isolates representative of currently circulating epidemic viruses (Fig. 1). However, a discord remains between these findings and historical evidence that suggests that the stability of IV in the environment can be influenced by RH (4, 9–14). We now provide a more refined explanation of the relationship between IV stability and RH that considers other factors, including virus strain background and time. We found that RH-mediated decay of seasonal IV varies with virus strain and propagation method, suggesting a role for virus- and host-specific factors in the maintenance of stability in the environment. To lend further support to this model, we have also found that the persistence of infectious virus in droplets over extended periods of time varies with RH and virus strain background.

Among seasonal IV, we identified differences between isolates of IBV and H3N2. Both viruses were stable in fine aerosols (Fig. 1), but IBV decayed significantly more than H3N2 in stationary droplets at 55% RH based on our statistical tests. Important distinctions between these two experiments are that (i) IV aerosolized into the drum were supplemented with ASL from HBE cells rather than being grown in HBE cells and that (ii) the viral aerosols were exposed to each RH for only 1 h in the rotating drum. These results, together with data from our previously published work (15), indicate that seasonal IV have the potential to remain highly stable and infectious while suspended in the atmosphere and also that aerosol or droplet size may be an important determinant of viral stability in the environment.

Among the IV exposed to a range of RH conditions in stationary droplets, all viruses responded to RH with generally more stability at low and high RH and the most decay under midrange conditions. However, the degrees of decay at midrange RH differed among the strains. Previous studies exploring the levels of stability of IAV and IBV smeared onto plastic (37) and banknotes (38) indicated that IAV tend to be more stable than IBV at midrange RH, but variations in ambient RH, virus suspension medium, mode of surface deposition, and surface material make direct comparisons difficult. In our study, virus strain and propagation method affected the degree to which each virus decayed under these conditions. Growth in HBE cells protected pre- and post-2009 pandemic H1N1 viruses from decay at midrange RH (Fig. 2A; see also Fig. S3 in the supplemental material). In contrast, we found that HBE cell-grown Perth H3N2 (Fig. 2B) and IBV (Fig. 3) decayed similarly to their MDCK cell-grown counterparts, although IBV may be more sensitive to RH than the seasonal IAV tested here. Much less is known about transmission of IBV than about that of IAV, although its spread may be driven primarily by children through direct contact (33). A recent study identified a correlation between IAV transmission efficiency and persistence of aerosolized virus infectivity in a ferret model (39). Further studies will be required to determine whether there is also a causal relationship between RH sensitivity and seasonal transmission cycles of epidemic IV.

We have shown that, similarly to the RH sensitivity results, the persistence of infectious IV over time varied with virus strain background and with RH. For example, the rate of decay of H1N1pdm in droplets did not increase for up to 16 h after deposition at midrange RH, while both Perth H3N2 and IBV continued to decay over time under all RH conditions tested (Fig. 4). Previous studies explored the persistence of IV, although never with viruses grown in primary human airway cells. Previous work showed that MDCK cell-grown H3N2 viruses can remain stable for up to 2 days following inoculation of a banknote, with persistence extended to days (IBV) or weeks (H3N2) under conditions of supplementation with human nasopharyngeal secretions (38). Importantly, both that previous study and our work demonstrated that infectious IV have the potential to persist on surfaces for extended periods of time. However, we have also shown that this persistence is closely linked to both atmospheric conditions and virus strain. The impact of other factors, such as deposition surface material, will also need to be considered in future studies of viruses in physiological droplets.

In contrast to seasonal IV, LPAI viruses, including avH6N1 and avH9N2, have not caused pandemic outbreaks in people (27). As with seasonal IV, infectious avian IAV have been shown to persist for days on various surfaces, although the RH at which these experiments were conducted was not noted (40). In our study, both of these viruses were highly sensitive to 55% RH in droplets following preparation in either MDCK or HBE cells (Fig. 5). Additionally, we showed that HBE cell-grown avH6N1, but not avH9N2, was significantly less stable in droplets at midrange RH than any of the other human-pathogenic seasonal IV tested (Fig. 6), suggesting that host species origin may contribute to the viral determinants of RH sensitivity. The interaction between IV and ASL under this critical midrange RH condition, including investigations of the role of ASL content in the environmental persistence of IV, will be the focus of future studies. The variations in the sensitivities of these seasonal IV and LPAI to midrange RH that we observed hints at a link between viral factors and the maintenance of viral infectivity outside the host which may impact the transmissibility of H6N1 and H9N2 LPAI viruses (28, 29). We do provide evidence for a link between HA receptor specificity and viral persistence in stationary droplets (Fig. S8), although further studies performed with additional mutants will be required to fully define this relationship. Other known determinants of IV transmissibility include viral surface protein function/stability and genomic background (36, 41–44). Integrational studies of these viral factors under various sets of environmental conditions will be required provide a framework for clarifying the mechanisms driving airborne transmission and may be useful for assessing the transmissibility or pandemic potential of emerging zoonotic IV without the need for animal model systems.

Here, we have clarified the relationship between RH and the stability of seasonal IV and LPAI strains resembling those that would be released into the environment from the airway of an infected person. This report provides a distinction between IV strains and virus stability in response to propagation method and specific environmental parameters. Aerosolized seasonal IV are highly resistant to RH-dependent decay, which suggests that removing them via increased air exchange rates, filtration, or UV germicidal irradiation may be critical to reducing the transmission of these viruses indoors. We found that RH is important for the stability of IV on surfaces but also that infectious viruses have the potential to persist on surfaces for hours in physiological droplets, reinforcing the need for surface decontamination in high-risk environments.

## MATERIALS AND METHODS

### Cells and viruses.

Primary HBE cells derived from human lung tissue were cultured at an air-liquid interface using an institutional review board-approved protocol (16). HBE ASL was collected for RH drum experiments by washing the apical surface of uninfected HBE cells with phosphate-buffered saline (PBS) as previously described (15). Madin-Darby canine kidney (MDCK) cells (ATCC) were cultured in Eagle’s minimal essential medium with 10% fetal bovine serum, l-glutamine, and penicillin-streptomycin. Influenza A viruses A/California/07/2009 (H1N1) pdm09 antiviral (AVR) reference virus M2—S31N NA—wild type (wt) (item number FR-458) and A/Brisbane/59/2007 (H1N1) FR-1 and influenza B virus B/Texas/02/2013 (Victoria lineage, cell-derived, FR-1302) were obtained through the International Reagent Resource, Influenza Division, World Health Organization Collaborating Center for Surveillance, Epidemiology, and Control of Influenza, Centers for Disease Control and Prevention (Atlanta, GA). Influenza A virus A/Perth/16/2009 (H3N2) was a generous gift from Zhiping Ye, Center for Biologics Evaluation and Research, Food and Drug Administration (Silver Spring, MD). LPAI influenza A viruses A/shorebird/DE Bay/230/2009 (H6N1) NR-45155 and A/shorebird/DE/127/2003 (H9N2) NR-45169 were obtained from BEI Resources, National Institute of Allergy and Infectious Diseases, National Institutes of Health (Bethesda, MD). Virus stocks were propagated in MDCK cells, harvested following detection of cytopathic effect (CPE), and clarified by low-speed centrifugation. HBE virus stocks were prepared by inoculation of each Transwell with 10^3^ TCID_50_/ml virus as previously described (17). Viruses and associated HBE ASL samples were harvested by pooling collected washes from the apical cell surface with PBS at 48 to 72 h postinfection (hpi), prior to the onset of CPE. LPAI replication kinetics in HBE cells were compared using three Transwells from each of two HBE cell lines at 1, 8, 24, and 48 hpi. IV titers were determined by TCID_50_ assay on MDCK cells according to the method of Reed and Muench (45).

### RH chamber for virus stability in stationary droplets.

Virus-laden droplets were exposed to seven RH conditions (23%, 33%, 43%, 55%, 75%, 85%, and 98%) in chambers conditioned for each RH using aqueous saturated salt solutions as previously described (14, 15). Briefly, chambers were housed inside a biosafety cabinet at room temperature, and temperature and RH were recorded during all experiments using an Onset HOBO temperature/RH logger. Ten 1-μl droplets were incubated in a 12-well tissue culture plate for the indicated time under each set of conditions. Residual virus was collected in 500 μl of L-15 tissue culture medium. Control samples consisting of 10 μl of virus suspension in an enclosed tube were incubated at ambient temperature within the same biosafety cabinet as the chamber during each experiment. The change in virus infectivity in response to RH is represented as log decay compared to the titer of control samples, as previously described (14). The rate of virus decay over time was quantified as Δ log decay, normalized to the average decay of each virus measured after 2 h under each set of RH conditions, where *x* is the indicated duration of incubation and *n* is the number of replicate samples collected at 2 h for each virus at each RH, as follows:

$$\log\;decay={\log\;decay}_{x\;h}-\frac{\Sigma ({\log\;decay}_{2\;h})}{n_{2\;h}}$$

### Statistical analysis.

Comparisons between levels of decay in MDCK cells versus HBE cells under all RH conditions were performed with a two-way ANOVA, with adjusted *P* values reported using Bonferroni’s multiple-comparison test. Significance was determined using a 95% confidence interval where *P* values of <0.05 are denoted by a single asterisk (*) and *P* values of <0.01 are denoted by double asterisks (**), unless otherwise indicated in the text. One-way ANOVA was used with a Tukey’s multiple-comparison test to compare levels of decay among three HBE patient cell lines for H1N1pdm, Perth H3N2, and IBV strains. Two-way ANOVA was used with Bonferroni’s multiple comparison’s test to compare levels of decay in three HBE patient cell lines for avH9N2 and avH6N1. Specific details regarding statistical tests are also provided in the figure legends. Statistical analyses were completed using GraphPad Prism version 8.0.0 software.

### Rotating RH drum used for analysis of virus stability in suspended aerosols.

Aerosolized viruses were exposed to selected RH conditions in a rotating, 27-liter aluminum drum housed within a biosafety cabinet, as previously described (15). Briefly, virus suspensions were aerosolized into the drum using a three-jet Collison nebulizer (BGI MRE-3) at a pressure level of 40 lb/in^2^. Target RH was achieved by adjusting the flow rates of the aerosol, dry air, and saturated air into the drum. Once RH reached equilibrium, an aerosol sample representing time zero was collected onto a gelatin filter at a flow rate of 2 liters/min for 15 min. The drum was then sealed, and aerosolized virus was incubated for 1 h, after which another aerosol sample was collected. The filters were dissolved into 3 ml of prewarmed L-15 medium, and IV titers were determined by TCID_50_ assay. Decay rates were corrected by RH-specific aerosol physical loss rates (15). Quantitative PCR (qPCR) was completed on RNA isolated from samples using a TaqMan RNA-to-*C_T_* 1-step kit (Thermo Fisher) (*C_T_*, threshold cycle) and a StepOnePlus real-time PCR system (Applied Biosystems). Viral RNA was detected using a probe against the IAV M gene segment (5′-FAM-TCAGGCCCCCTCAAAGCCGA-BHQ1-3′) (FAM, 6-carboxyfluorescein; BHQ1, black hole quencher 1).
