# Supplementary material for: Environmental Persistence of Influenza Viruses Is Dependent upon Virus Type and Host Origin
Source: mSphere. 2019 Aug 21;4(4):e00552-19. doi: 10.1128/mSphere.00552-19 (PMC6706471; doi:10.1128/mSphere.00552-19)
Supplement: TABLE S1 [file mSphere.00552-19-st001.docx]

**Supplemental Table S1. Influenza viruses passage history and references.**

| H1N1pdm | | |
| --- | --- | --- |
| Source | **Strain Reference** | **Virus Propagation History*^a^*** |
| Influenza Reagent Resource, Influenza Division, WHO Collaborating Center for Surveillance, Epidemiology, and Control of Influenza, Centers for Disease Control and Prevention, Atlanta, GA, USA | Influenza A Virus, A/California/07/2009 (H1N1)pdm09 Antiviral Resistance (AVR) – Reference Virus M2: S31N NA: wild type (wt), FR-458 | ePx, cP3 |
| α2,3 H1N1pdm | | |
| Generous gift from Dr. Kanta Subbarao (formerly NIH) | A/California/07/09 (7:1) HA α2,3 | ep2, ep1 |
| Bris H1N1 | | |
| Source | **Strain Reference** | **Virus Propagation History*^a^*** |
| Influenza Reagent Resource, Influenza Division, WHO Collaborating Center for Surveillance, Epidemiology, and Control of Influenza, Centers for Disease Control and Prevention, Atlanta, GA, USA | Influenza A Virus, A/Brisbane/59/2007 (H1N1), FR-1 | eP2, eP2, eP3, cP2 |
| Perth H3N2 | | |
| Source | **Strain Reference** | **Virus Propagation History*^a^*** |
| Generous gift from Dr. Zhiping Ye (Center for Biologics Evaluation and Research, FDA) | A/Perth/16/2009 (H3N2) | ePx, cP3 |
| IBV | | |
| Source | **Strain Reference** | **Virus Propagation History*^a^*** |
| Influenza Reagent Resource, Influenza Division, WHO Collaborating Center for Surveillance, Epidemiology, and Control of Influenza, Centers for Disease Control and Prevention, Atlanta, GA, USA | Influenza B Virus, B/Texas/02/2013 (Victoria Lineage), Cell-Derived, FR-1302 | cP2 |
| avH6N1 | | |
| Source | **Strain Reference** | **Virus Propagation History*^a^*** |
| BEI Resources, NIAID, NIH | Influenza A Virus, A/shorebird/Delaware Bay/230/2009 (H1N1), NR-45155 | ePx, cP3 |
| avH9N2 | | |
| Source | **Strain Reference** | **Virus Propagation History*^a^*** |
| BEI Resources, NIAID, NIH | Influenza A Virus, A/shorebird/Delaware Bay/127/2003 (H9N2), NR-45169 | ePx, cP3 |

*^a^*The total number of prior egg passages (eP) and cell passages (cP) of each virus are listed in the table.
