# Supplementary material for: Environmental Persistence of Influenza Viruses Is Dependent upon Virus Type and Host Origin
Source: mSphere. 2019 Aug 21;4(4):e00552-19. doi: 10.1128/mSphere.00552-19 (PMC6706471; doi:10.1128/mSphere.00552-19)
Supplement: TABLE S2 [file mSphere.00552-19-st002.docx]

**Supplemental Table S2. Influenza viruses and corresponding HBE cell lines used for RH chamber experiments.**

| H1N1pdm | | | |
| --- | --- | --- | --- |
| Time (h) | **%RH** | **Cell Line** | **Cell Phenotype*^a^*** |
| 2 | 23, 33, 43, 55, 75, 85, 98 | HBE 0133 | MCTD |
| 2 | 23, 43, 75, 98 | HBE 0147 | IPF |
| 2 | 23, 43, 75, 98 | HBE 0154 | CORE |
| 8, 16 | 23, 43, 75, 98 | HBE 0154 | CORE |
| 8, 16 | 23, 43, 75, 98 | HBE 0167 | COPD |
| 2, 8, 16 | 23 | HBE 0167 | COPD |
| 8, 16 | 75 | HBE 0176 | CORE |
| α2,3 H1N1pdm | | | |
| Time (h) | **%RH** | **Cell Line** | **Cell Phenotype** |
| 2 | 23, 33, 43, 55, 75, 85, 98 | HBE 0231 | IPF |
| 2 | 23, 33, 43, 55, 75, 85, 98 | HBE 0235 | CORE |
| 2 | 23, 33, 43, 55, 75, 85, 98 | HBE 0237 | IPF |
| Bris H1N1 | | | |
| Time (h) | **%RH** | **Cell Line** | **Cell Phenotype** |
| 2 | 23, 33, 43, 55, 75, 85, 98 | HBE 0207 | CORE |
| Perth H3N2 | | | |
| Time (h) | **%RH** | **Cell Line** | **Cell Phenotype** |
| 2 | 23, 33, 43, 55, 75, 85, 98 | HBE 0133 | MCTD |
| 2 | 23, 33, 43, 55, 75, 85, 98 | HBE 0167 | COPD |
| 8, 16 | 23, 43, 75, 98 | HBE 0147 | IPF |
| 2 | 23, 43, 75, 98 | HBE 0154 | CORE |
| 8, 16 | 23, 43, 75, 98 | HBE 0154 | CORE |
| 2, 8, 16 | 23, 43 | HBE 0154 | CORE |
| 8, 16 | 75 | HBE 0167 | COPD |
| IBV | | | |
| Time (h) | **%RH** | **Cell Line** | **Cell Phenotype** |
| 2 | 23, 33, 43, 55, 75, 85, 98 | HBE 0133 | MCTD |
| 2 | 23, 33, 43, 55, 75, 85, 98 | HBE 0167 | COPD |
| 2 | 23, 33, 43, 55, 75, 85, 98 | HBE 0207 | CORE |
| 8, 16 | 23, 43, 75, 98 | HBE 0147 | IPF |
| 8, 16 | 23, 43, 75, 98 | HBE 0167 | COPD |
| 16 | 75, 98 | HBE 0167 | COPD |
| 8, 16 | 23, 43, 75, 98 | HBE 0207 | CORE |
| avH6N1 | | | |
| Time (h) | **%RH** | **Cell Line** | **Cell Phenotype** |
| 2 | 23, 33, 43, 55, 75, 85, 98 | HBE 0195 | CORE |
| 2 | 23, 33, 43, 55, 75, 85, 98 | HBE 0206 | IPF |
| 2 | 23, 33, 43, 55, 75, 85, 98 | HBE 0204 | PF |
| avH9N2 | | | |
| Time (h) | **%RH** | **Cell Line** | **Cell Phenotype** |
| 2 | 23, 33, 43, 55, 75, 85, 98 | HBE 0195 | CORE |
| 2 | 23, 33, 43, 55, 75, 85, 98 | HBE 0206 | IPF |
| 2 | 23, 33, 43, 55, 75, 85, 98 | HBE 0204 | PF |

*^a^*Cell phenotypes correspond to lung tissue samples from which the cell lines were derived: MCTD (mixed connective tissue disease), IPF (idiopathic pulmonary fibrosis), COPD (chronic obstructive pulmonary disease), PF (pulmonary fibrosis), and CORE (wildtype).
